# Hormonal control of promoter activities of *Cannabis* *sativa* prenyltransferase 1 and 4 and salicylic acid mediated regulation of cannabinoid biosynthesis

**DOI:** 10.1038/s41598-023-35303-4

**Published:** 2023-05-27

**Authors:** Lauren B. Sands, Samuel R. Haiden, Yi Ma, Gerald A. Berkowitz

**Affiliations:** 1 Department of Plant Science and Landscape Architecture, Agricultural Biotechnology Laboratory, University of Connecticut, Storrs, CT 06269-4163 USA; 2 Present Address: SafeTiva Labs, Westfield, MA 01085 USA

**Keywords:** Plant sciences, Plant hormones, Plant molecular biology, Secondary metabolism

## Abstract

*Cannabis* *sativa* aromatic prenyltransferase 4 (*CsPT4*) and 1 (*CsPT1*) have been shown to catalyze cannabigerolic acid (CBGA) biosynthesis, a step that rate-limits the cannabinoid biosynthetic pathway; both genes are highly expressed in flowers. *CsPT4* and *CsPT1* promoter driven β-glucuronidase (GUS) activities were detected in leaves of cannabis seedlings, and strong *CsPT4* promoter activities were associated with glandular trichomes. Hormonal regulation of cannabinoid biosynthetic genes is poorly understood. An in silico analysis of the promoters identified putative hormone responsive elements. Our work examines hormone-responsive elements in the promoters of *CsPT4* and *CsPT1* in the context of physiological responses of the pathway to the hormone in planta. Dual luciferase assays confirmed the regulation of promoter activities by the hormones. Further studies with salicylic acid (SA) demonstrated that SA pretreatment increased the expression of genes located downstream of the cannabinoid biosynthetic pathway. The results from all aspects of this study demonstrated an interaction between certain hormones and cannabinoid synthesis. The work provides information relevant to plant biology, as we present evidence demonstrating correlations between molecular mechanisms that regulate gene expression and influence plant chemotypes.

## Introduction

*Cannabis* *sativa,* an herbaceous flowering plant, is emerging as a key player in the future of medicine^1^. Along with terpenes, the production of cannabinoids by the translation products of the cannabinoid biosynthetic pathway genes (and factors that affect their expression) determine the medicinal value of the plant^2^. The bulk of cannabinoid production occurs predominantly in unfertilized female cannabis flowers and increases substantially as these female flowers undergo developmental changes during maturation^3^. There is a lack of understanding of how these genes are regulated throughout the flowering period, despite the potential importance of this regulation in terms of the impact that cannabinoid production has on the value of this crop plant.

The precursors of cannabinoids originate from two pathways: the polyketide pathway and the plastidial 2-C-methyl-d-erythritol 4-phosphate (MEP) pathway^4^. The polyketide pathway gives rise to olivetolic acid (OA), while the MEP pathway gives rise to geranyl diphosphate (GPP). GPP is the precursor for monoterpenes, and it is also involved in the step of the cannabinoid biosynthetic pathway that produces cannabigerolic acid (CBGA)^2^. Although GPP affects plant phenotype in a number of ways (for example, impacting growth and development in addition to monoterpene synthesis in *Camelina* *sativa*^5^) in cannabis it serves as a substrate for the two aforementioned biosynthetic pathways. How, and if its relative use in these two pathways is regulated is not understood^6^*.* Aromatic prenyltransferase (PT) enzymes are involved in the synthesis of many specialized metabolites. In cannabis, specific plastid membrane-localized PT isoforms 1 and 4 are candidates to catalyze the alkylation of GPP and OA to form CBGA^7^. It should be noted that PTs generate a broad range of specialized metabolites in numerous plant species^8^, but here we focus on the production of CBGA in cannabis. CBGA is the precursor to many end-point cannabinoids, a few key ones including cannabidiolic acid (CBDA), tetrahydrocannabinolic acid (THCA), and cannabichromenic acid (CBCA)^4^.

Previous studies have focused on THCA synthase (THCAS) as the rate-limiting enzyme in the production of THCA, likewise with CBDAS and CBDA^9,10^. However, conclusions from recent work from our lab and others have determined that the correlation between *THCAS* gene expression and THCA content is poor, and attention should be paid to other enzymes in the cannabinoid biosynthetic pathway^11,12^. Our prior studies further identified the production of CBGA as rate-limiting terminal cannabinoid synthesis^12^.

Research indicates that geranyl diphosphate synthase (*GPPS*) rate-limits the production of monoterpenes in various plant species, such as orchids^13^. Monoterpenes can be synthesized from the largely conserved MEP pathway in plants^14^, indicating that *GPPS* is likely rate-limiting monoterpene production in cannabis, while simultaneously being involved in cannabinoid biosynthesis. CsPT1 and CsPT4 enzymes utilize GPP, synthesized by GPPS, to produce CBGA^15^. This has led us to believe that studying the regulation of the expression of genes facilitating CBGA production will enhance our understanding of the genetic modulation of cannabinoid production.

Both CsPT1 and CsPT4 have been demonstrated to catalyze the alkylation of OA and GPP to form CBGA^15,16^. They both use GPP as the prenyl donor while accepting different substrates. CsPT1 is promiscuous and can accept multiple substrates, including OA, while CsPT4 only accepts OA. A study conducted by Page et al. concluded that CsPT1 exhibits a Km for OA at 60 mM^16^, while Luo et al. determined that CsPT4 has a Km of 6.72 μM for OA^15^. Gülck et al. demonstrated in yeast and *Nicotiana* *benthamiana* that only CsPT4 can make CBGA but not other CsPT genes including CsPT1^17^. These findings indicate that CsPT4 is more likely to convert OA and GPP into CBGA in cannabis female flowers, however, more research on these enzymes is required to gain a better understanding of their functions.

This research focuses on the molecular signals that initiate and regulate cannabinoid production, which originates with trichome development. These processes depend on hormonal signals, transcriptional activators, or repressors^18^. There are many hormones involved in plant development and regulation in response to environmental signals (abiotic and biotic stress, for example)^19^. We included salicylic acid (SA), gibberellic acid (GA), auxin (NAA), abscisic acid (ABA), cytokinin (trans-Zeatin), ethylene and jasmonic acid (JA) in the analysis. The goal of this research was to understand how genes encoding cannabinoid biosynthetic enzymes that potentially rate-limit cannabinoid synthesis are regulated through hormone networks. Both *CsPT1* and *CsPT4* were highly expressed in female flowers and high promoter activity was associated with the location of glandular trichomes. *CsPT1* and *CsPT4* promoters were activated by various hormones identified by the bioinformatic analysis of hormone responsive elements. The findings of this research can increase knowledge regarding enzyme functionality in cannabis and provide genetic information to the industry for breeding of new cannabis varieties with enhanced cannabinoid production.

## Results

### Expression patterns of *CsPT1* and *CsPT4*

**Figure 1:**
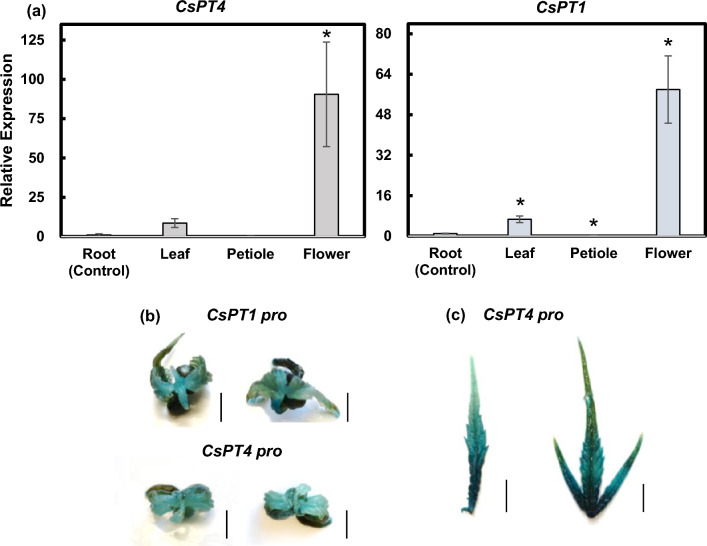
*CsPT4* and *CsPT1* expression pattern in cannabis tissues. (**a**) Transcript levels of *CsPT4* and *CsPT1* in various tissues, including root, leaf, petiole, and flower. Expression levels of each tissue were compared to the root. Student’s *t*-test was utilized for calculating statistical significance of means separation; *indicates *p* ≤ 0.05 (n = 3). (**b**,**c**) Transient transfection of *CsPT4* *pro* and *CsPT1* *pro* driven GUS reporter in cannabis seedlings (**b**) and sugar leaves (**c**). Scale bars represent 5 mm.

Real-time quantitative PCR (qPCR) was conducted on different tissues in the female cannabis plant, and root expression was used as the control, to determine in which tissue the expression of these genes is highest. Results showed that both *CsPT1* and *CsPT4* were highly expressed in the flowers, and their transcripts were also detected in the leaves, suggesting their primary function is in the flower tissue (Fig. 1). We examined the locations of promoter activities in hemp seedlings and sugar leaves using a GUS reporter. GUS activity driven by the *CsPT1* and *CsPT4* promoters (*CsPT1* *pro* and *CsPT4* *pro*) was analyzed.

The 1500 bp region upstream of the coding sequence (CDS) of *CsPT1* and the 600 bp regions upstream of the CDS of *CsPT4* were individually cloned to create a *CsPT* *pro:*GUS construct (Supplementary Fig. 1 and Supplementary Table 2). Activities of *CsPT1* *pro* and *CsPT4* *pro* were observed in the young leaves of cannabis seedlings (Fig. 1b). In addition, the activity of *CsPT4* *pro* was much stronger toward the base of the sugar leaves, where the capitate stalked glandular trichomes are predominantly developed (Fig. 1c and Supplementary Fig. 2), further indicating the main function of *CsPT4* is in these glandular trichomes.

### In silico analysis of *CsPT1 and CsPT4* promoters

As shown in Fig. 1b,c, the 600 bp region of *CsPT4* *pro* we cloned was sufficient to drive strong expression of coding sequences cloned downstream of this promoter region*.* Analysis using PLACE and PlantCARE, the online query software for cis-acting regulatory element analyses, revealed many putative responsive elements in the cloned promoter fragments of *CsPT1* and *CsPT4*. These elements are conserved sequences which have been demonstrated, in other organisms, to bind to transcription factors related to various environmental cues such as stress, wounding, drought, and hormone signaling^20^.

The hormone responsive elements for both *CsPT1* *pro* and *CsPT4* *pro* are listed in Table 1, and contextually visualized in Supplementary Figs. 3 and 4. The in silico analysis showed that in *CsPT4* *pro*, there are three responsive elements for cytokinin, one for SA, one for GA, one for ethylene, and one for ABA. The analysis also revealed that in *CsPT1* *pro*, there are five GA responsive elements, two SA, six auxin, and three ABA responsive elements. MeJA signaling is vital for glandular trichome formation and specialized metabolite biosynthesis^21–26^. However, no JA responsive elements were found in either promoter. It is possible that JA may not activate *CsPT1* and *CsPT4*.

**Table 1:** In silico identification of hormone responsive elements of *CsPT1* and *CsPT4* promoters.

Promoter	*CsPT1* (1500 bp region)	*CsPT4* (600 bp region)
Cytokinin (ZR)^a^	0	3
Gibberellin (GA_3_)	5	1
Abscisic acid (ABA)	3	1
Salicylic acid (SA)	2	1
Ethylene (ACC)^a^	0	1
Auxin (NAA)^a^	3	0

The promoters were analyzed using PLACE and PlantCARE online programs.

^a^ZR, trans-zeatin riboside; ACC, 1-aminocyclopropane-1-carboxylic acid, the direct ethylene precursor; NAA, 1-naphthaleneacetic acid.

### *CsPT4 pro* exhibits a strong response towards various hormones

To understand hormonal regulation of the *CsPT* genes, Dual Luciferase (LUC) Reporter Assay (DLR) was performed in Arabidopsis protoplasts. The same promoter sequences used for the GUS assay were cloned to create *CsPT* *pro:*LUC constructs (Supplementary Fig. 1). The *CsPT4* *pro* that was cloned contains three putative cytokinin responsive elements; *CsPT4* *pro* was predicted to have no auxin responsive elements. Therefore, NAA was used as a negative control. The activities of LUC driven by the promoters were measured after hormone treatment using luminescence.

**Figure 2:**
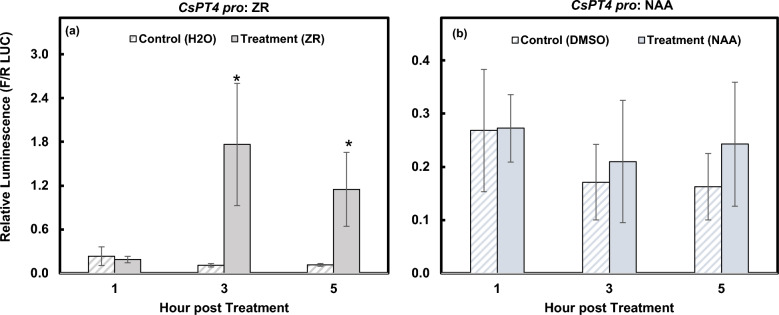
Responses of *CsPT4* *pro* to ZR and NAA in Arabidopsis protoplasts. DLR results showed the activity of the *CsPT4* *pro* indicated by relative luminescence, calculated by a ratio of firefly LUC to *Renilla* LUC. Responses to ZR (**a**) and NAA (**b**) at 1, 3, and 5 hpt are shown. A Student’s *t*-test was used to compare relative luminescence of treated protoplasts to the solvent control at each time point. This method of statistical analysis was repeated for each DLR assay (Figs. 3, 4, 5). *indicates *p* ≤ 0.05 (n = 3).

At 3 and 5 h post treatment (hpt) of ZR, *CsPT4* *pro* activated the luciferase reporter at significant levels (Fig. 2a). This result is consistent with our aforementioned in silico analysis that cytokinin may regulate *CsPT4* expression. Because there are no predicted auxin responsive elements in *CsPT4* *pro*, it is expected that there would be no increased luciferase activity upon auxin treatment. Following application of NAA, there was no increased activity measured from *CsPT4* *pro*, indicating that auxin may not modulate *CsPT4* transcription in Arabidopsis protoplasts (Fig. 2b).

**Figure 3:**
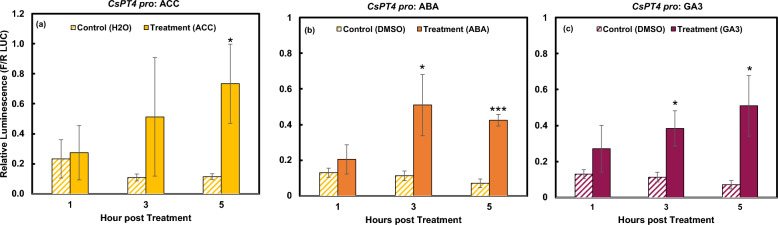
Responses of *CsPT4* *pro* to ACC, ABA, and GA. DLR results display the activation of *CsPT4* *pro* over 5 h indicated by relative luminescence, calculated by a ratio of firefly to *Renilla* LUC. Significant activation seen from ACC (**a**), ABA (**b**), and GA_3_ (**c**). *indicates *p* ≤ 0.05. ***indicates *p* ≤ 0.001 (n = 3).

In addition, the 600 bp *CsPT4* promoter region contains one ethylene, one ABA, and one GA putative responsive element. At 5 hpt with ACC (causing ethylene rise), *CsPT4* *pro* was able to be activated at significant levels (Fig. 3a). In response to ABA and GA_3_, *CsPT4* *pro* was activated at 3 and maintained at 5 hpt (Fig. 3b,c). These three hormones had similar levels of *CsPT4* *pro* activation. The results indicate that *CsPT4* can be involved in different hormonal signaling pathways; this supports the hypothesis that hormones may coordinate to control glandular trichome development and cannabinoid biosynthesis in cannabis female flowers^27–30^.

### Auxin and ABA activate *CsPT1 pro*

**Figure 4:**
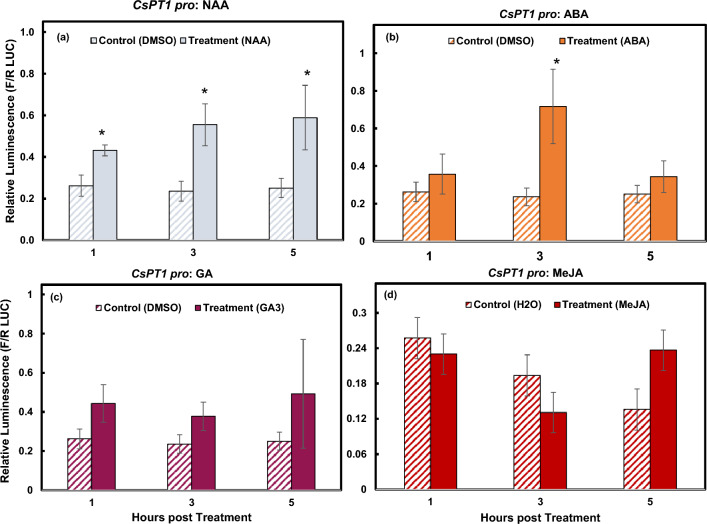
Responses of *CsPT1* *pro* to ABA, NAA, GA, and MeJA**.** Dual luciferase assay results displaying activity of *CsPT1* *pro* at 1, 3, and 5 hpt indicated by relative luminescence. Significant activation was measured from NAA (**a**) as well as ABA (**b**). No activation of *CsPT1* *pro* was detected in response to GA_3_ (**c**) or MeJA (**d**). *indicates *p* ≤ 0.05. **indicates *p* ≤ 0.01 (n = 3).

The 1500 bp region of *CsPT1* *pro* contains three auxin, three ABA, and five GA putative responsive elements. Following treatment from NAA, *CsPT1* *pro* was activated significantly as early as 1 hpt. The activity was slightly higher at 3 hpt and retained at 5 hpt (Fig. 4a). ABA was able to induce significant activation of *CsPT1* *pro* only at 3 hpt and the activity returned to the basal level 5 hpt (Fig. 4b). Treatment with GA_3_ prompted slight increases in relative luminescence, however there was no significant difference compared to the solvent control at each time point (Fig. 4c).

MeJA is a major hormone involved in plant defense responses to stress stimuli^31–33^. In a previous study, we showed that MeJA foliar spray increased cannabinoid production^12^. Although no MeJA responsive elements were found in *CsPT1* *pro*, the response of *CsPT1* *pro* towards MeJA was still examined. Interestingly, there was slight decrease of luminescence 3 hpt and then slight increase of luminescence 5 hpt (Fig. 4d). However, there was no significant difference observed at either time point. MeJA could regulate other cannabinoid biosynthetic genes or promote glandular trichome morphogenesis, resulting in higher cannabinoid accumulation.

### *CsPT1 pro* and *CsPT4 pro* are responsive to SA

**Figure 5:**
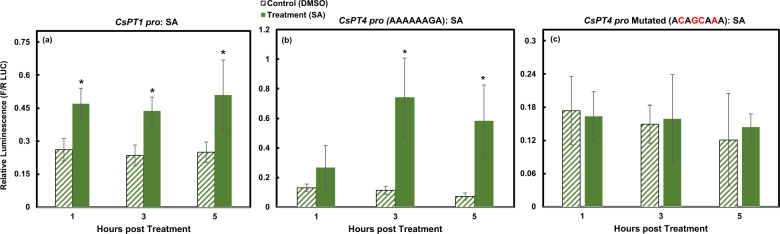
SA activated *CsPT1* *pro* and *CsPT4* *pro*. Dual luciferase assay results displaying activation of *CsPT1* *pro* (**a**) and *CsPT4* *pro* (**b**) across 5 h indicated by relative luminescence. Mutated *CsPT4* *pro* (**c**) did not respond to SA. *indicates *p* ≤ 0.05 (n = 3).

*CsPT1* *pro* and *CsPT4* *pro* were examined collectively for their response to SA. Both promoters were activated after treatment with SA (Fig. 5). *CsPT1* *pro* was activated sooner at 1 hpt and the activity was retained at similar levels from 1 to 5 hpt (Fig. 5a). *CsPT4* *pro* was not activated until 3 hpt with a slight decrease at 5 hpt (Fig. 5b). Intriguingly, the increased activity of *CsPT4* *pro* was substantially higher than that of *CsPT1* *pro* despite it containing one more SA Responsive Element (SARE), suggesting a greater demand of *CsPT4* transcripts after SA elicitation.

To further understand whether the identified putative SARE in *CsPT4* *pro* is truly responsive to SA, site-directed mutagenesis was performed to introduce mutations in the sequence. Four base pairs were mutated to diminish or eliminate responses of *CsPT4* *pro* to SA elicitation (Fig. 5c and Supplementary Fig. 5). The results revealed that the mutated *CsPT4* *pro* was no longer responsive to SA elicitation (Fig. 5c), indicating that the identified SARE in *CsPT4* *pro* is a *bona* *fide* *cis*-acting element involved in SA signaling.

### SA induces certain cannabinoid biosynthetic pathway genes

SA root drench at 1 mM was administered to flowering cannabis plants. This resulted in altered expression of six genes involved in the cannabinoid biosynthetic pathway. The genes examined include tetraketide synthase (*TKS*), olivetolic acid cyclase (*OAC*), geranyl diphosphate synthase small subunit (*GPPS* *ssu*), *CBDAS*, *CsPT1* and *CsPT4* (primer sequences are listed in Supplementary Table 2). We were able to identify predicted SAREs in the promoters of *TKS*, *OAC*, *CBDAS*, *CsPT1*, and *CsPT4*.

**Figure 6:**
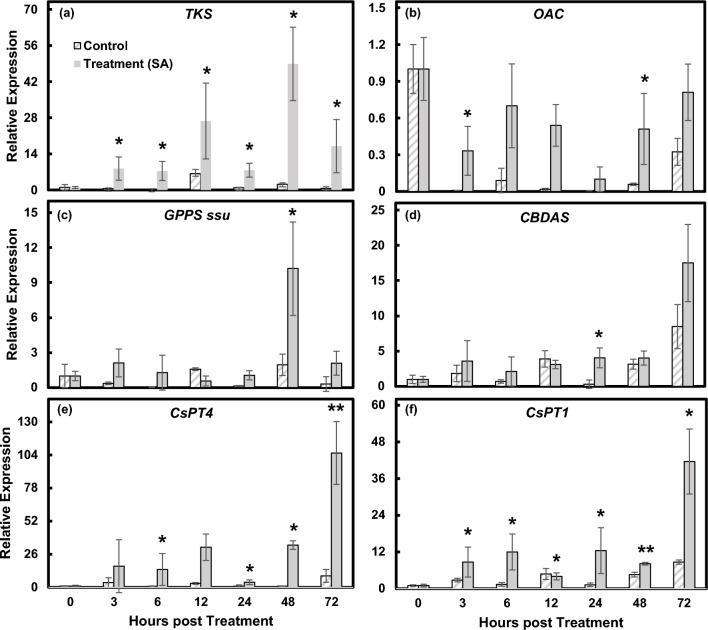
Real time PCR analysis of cannabinoid biosynthetic genes following SA treatment over a 72-h period. Graphs shown include genes: (**a**) *TKS*, (**b**) *OAC*, (**c**) *GPPS* *ssu*, (**d**) *CBDAS* (**e**) *CsPT4* and (**f**) *CsPT1*. Results were averaged from four biological replicates. Statistical analysis was performed in comparison to DMSO control at each time point using Student’s *t*-test. *indicates *p* ≤ 0.05. **indicates *p* ≤ 0.01. ***indicates *p* ≤ 0.001 (n = 4).

*TKS* expression levels were significantly upregulated at every examined time point post treatment. *TKS* expression reached the peak 48 hpt and dropped 72 hpt but still measured significantly higher than the control (Fig. 6a). *TKS* is a type III polyketide synthase and works together with *OAC* to produce olivetolic acid from hexanoyl-CoA and malonyl-CoA. Interestingly, *OAC* transcript levels were reduced compared to time 0, however, at each time point, the treated samples still showed higher expression levels compared to the control (Fig. 6b).

It has been reported that the ratio of the small (ssu) to large subunit of GPPS determines the amount of GPP synthesized^34,35^, therefore *GPPS* *ssu* was chosen for the analysis. *GPPS* *ssu* expression did not significantly increase until 48 hpt and then drastically dropped at 72 hpt (Fig. 6c). In general, *CBDAS* was not upregulated by SA except for 24 hpt, which was mainly due to the decreased expression in the control samples; the expression levels were similar to other time points except for 72 hpt (Fig. 6d), suggesting that SA may not be responsible for inducing *CBDAS* expression. Interestingly, *CBDAS* transcripts increased at 72 hpt compared to earlier time points in the control samples. SA increased *CBDAS* expression to a higher level at 72 hpt compared to the control, but no significant difference was observed (Fig. 6d).

*CsPT* gene expression can be regulated by SA. Figure 6e showed that both *CsPT1* and *CsPT4* transcript levels started to increase 3 or 6 hpt and were maintained at similar levels during the first 48 hpt, except for *CsPT4* at 24 hpt and *CsPT1* at 12 hpt. At 72 hpt, both genes increased substantially with *CsPT4* being upregulated around 100-fold compared to the control at that time. The gene expression analysis is consistent with results obtained from DLR assay (Fig. 5a,b).

## Discussion

### CsPT1 vs. CsPT4: which enzyme plays a greater role for CBGA synthesis?

Both CsPT4 and CsPT1 have been demonstrated to produce CBGA from OA and GPP (in heterologous systems such as yeast) but more recent findings have shown that CsPT4 is the only enzyme that synthesizes CBGA^6,15,16^; CsPT1 failed to generate CBGA in in vitro assays in yeast and *Nicotiana* *benthamiana*^6,15^. OA and analogs can be accepted as substrates for CsPT4, while CsPT1 is promiscuous and has multiple aromatic substrates, which was summarized in de Bruijn et al.^7^.

Previous studies were focused on in vitro assays of the abilities of CsPT1 and CsPT4 enzymes in synthesizing CBGA. There was no study on the hormone regulated transcriptional control of these genes and how hormonal regulation could be associated with cannabinoid biosynthesis. *CsPT4* and *CsPT1* are both abundantly expressed in the flowers (Fig. 1a), and the promoters of *CsPT1* and *CsPT4* are active in the leaves of young seedlings, with stronger activities in the glandular trichomes. The results suggest that both genes may be evaluated as important enzymes facilitating cannabinoid production. The high expression level of *CsPT1* in the flowers and the upregulation by SA suggests that CsPT1 may be involved in the biosynthesis of cannabinoids under certain conditions or the biosynthesis of other specialized metabolites in the glandular trichomes. Because all the assays on CsPT1 enzyme activity were conducted in other organisms^15–17^, it is also possible that CsPT1 functions differently in cannabis plants.

CsPT4 has shown about 10,000 times greater Km (60 mM for CsPT1 and 6.72 μM for CsPT4) to OA than CsPT1^15,16^. Although both CsPT1 and CsPT4 are highly expressed in the flowers, the Km implies that CsPT4 is more likely to be the enzyme that binds to OA for CBGA biosynthesis. In addition, when administered the necessary OA in engineered yeast strains, CsPT4 was able to produce CBGA while CsPT1 failed to do so^15^. CsPT1 only showed enzymatic activity in *E.* *coli* but not in eukaryotic systems, such as yeast and N. benthamiana^17^. Our study also indicates that the promoters of CsPT1 and CsPT4 are responsive to different hormones, except for ABA and SA (Figs. 2, 3, 4, 5). Our findings suggest that CsPT1 and CsPT4 could be involved in different biosynthesis pathways. While studying these enzymes in other biological systems demonstrated some evidence of the functions of these two enzymes in the biosynthesis of CBGA, it is still not fully understood how the enzymes are acting in glandular trichomes on cannabis female flowers. Further genetic analysis using CsPT mutants, techniques for overexpression, gene silencing, or knock-out of the genes, will provide more explicit evidence on the involvement of these two CsPT enzymes in CBGA biosynthesis in vivo; it should be noted that at present, cannabis is recalcitrant to transformation and these studies could be pursued with a more robust transformation protocol than those available at present.

### Hormone regulation of *CsPT* promoters

Specialized metabolite biosynthesis is regulated by plant hormones^26,36^. Results from this research demonstrated that *CsPT4* *pro* was responsive towards cytokinin, ABA, ethylene, SA, and GA, while *CsPT1* *pro* was only activated following treatment with auxin, ABA, and SA (Figs. 2, 3, 4, 5), providing hints on the association between hormone signaling and cannabinoid biosynthesis.

ABA could activate both promoters in our study (Figs. 3b, 4b). ABA plays essential roles in the regulation of floral development and plant responses to abiotic stress^37^. Our previous study showed that both *CsPT1* and *CsPT4* were upregulated during female flower maturation and associated with glandular trichome development^12^. Previous studies demonstrated that ABA alone did not affect flower sex determination, however, ABA inhibited indole-3-acetic acid (IAA) induced female flower formation and impaired GA induced male flower development^38,39^. ABA treatment increased THC concentration in both leaf and female flower tissues^30^ and has been shown to promote trichome formation^40^. The activation of the promoters of *CsPT1* and *CsPT4* by ABA suggests that ABA may also play important roles in glandular trichome development and cannabinoid biosynthesis during female flower maturation in cannabis plants.

The promoter of *CsPT4* was also activated by cytokinin, ethylene, and GA but not auxin (Figs. 2, 3). These hormones have been shown to be important for sex determination during floral development in dioecious or gynoecious plants^41–44^. Cytokinin production in roots was shown to be essential for female sex determination in hemp plants generated from shoot cuttings of young plants^45^. Ethylene was shown to promote female flower development in industrial hemp^38,46–48^. Exogenous GA application induced male flower formation on female cannabis plants, which was inhibited by ABA^39^. GA signaling associated genes were also shown to be upregulated in induced-male (on female flowers) and male flowers compared to female flowers^44^.

In our study, NAA did not activate *CsPT4* *pro* (Fig. 4a), even though auxin (IAA) was shown to be involved in the induction of female flowers^38^. Promoters of *CsPT1* and *CsPT4* were activated by hormones important for both female and male flower induction, indicating that *CsPT1* and *CsPT4* could be active in both female and male flowers. These findings also imply that cannabinoid biosynthesis involves the interaction of different hormonal pathways that are essential for female flower induction and development. Monitoring of hormone content during cannabis flower development will enable us to further understand how these hormones function in cannabinoid or other specialized metabolite biosynthesis.

The DLR assays for hormone regulation of *CsPT1* *pro* and *CsPT4* *pro* were conducted using Arabidopsis protoplasts. Although many transcription factors are conserved among different plant species, there are also many with distinct functions in certain species. The results we observed in Arabidopsis protoplasts may be inconsistent or even contradictory. It would be interesting and important to further investigate the functions of these hormones in cannabis plants. Our findings still provide insights and clues to the hormonal modulation of glandular trichome development and cannabinoid biosynthesis in cannabis female flowers.

### SA plays important roles in the cannabinoid biosynthetic pathway

SA is primarily involved in plant defense responses to abiotic and biotic stresses. It is also an efficient elicitor for specialized metabolite biosynthesis^49–51^. As a plant undergoes abiotic or biotic stress, SA is produced at higher concentrations, in turn activating defense signaling pathways^52^. Cannabinoids are known to be toxic to cells and must be exported to the secretory cavity of glandular trichomes^53,54^, where they may act as a defense mechanism against herbivory, fungi and bacteria^55^. Our previous work showed that MeJA increased cannabinoid production and this hormone is also involved in defense pathways^12,56^. However, no JA responsive element was identified in the *CsPT* promoters.

In this report, we focused on the effects of SA on the cannabinoid biosynthetic pathway. One SARE in the cloned 600 bp of *CsPT4* pro was identified. After searching a broader region of the promoter, one more SARE was also identified. The one SARE in the 600 bp region was able to respond to SA application, which was further confirmed in that the 600 bp of *CsPT4* pro with a mutated SARE failed to be activated by SA (Fig. 5b). Moreover, the expression of *CsPT4* was substantially increased by root drench of SA (Fig. 6e). In addition, SA also increased other cannabinoid biosynthetic gene expression levels including *TKS,* *CBDAS,* *CsGPPS* *ssu,* and *CsPT1* (Fig. 6).

Different SA approaches were utilized, as seen in other studies, to evaluate effects on cannabis^19,28,57–61^. Foliar application of methyl salicylate (MeSA), which can be hydrolyzed to produce SA^62^, to flowering plants resulted in a 47% increase in CBDA content per total flower weight (Supplementary Fig. 6). Although no significant difference was observed, there was a trend of increased CBDA content, suggesting that components of the SA signaling pathway can potentially increase cannabinoid production. Future studies involving more biological replicates and more cannabinoid measurements, may result in significant increases in cannabinoid content following hormone treatments. SA has been shown to be involved in flowering in various plant species, such as Arabidopsis^19^, *Lemna* *pauciostate* (duckweed)^63^, and *Oryza* *sativa* (rice)^64^. SA was also demonstrated to increase glandular trichome size and density in several species^65,66^. Our findings provide strong evidence that SA can positively regulate cannabinoid biosynthesis.

## Experimental procedures

### Plant materials and growth conditions

Wild type (Col-0) Arabidopsis seeds were surface sterilized with bleach, then plated on agar with half-strength Murashige and Skoog (MS) salts (Caisson Labs, Logan, UT, USA), MES (adjusted to pH 5.7 with Tris), 1% sucrose, and 0.8% agar, and grown in a growth chamber with a day (80–100 μmol m^− 2^ s^− 1^ illumination)/night cycle of 16 h/8 h at 22 °C. 8 days after germination, the seedlings were transferred into potting mix and grown in a growth chamber with LED lights at 12 h light/12 h dark at 22 °C.

Samples, Fig. 1: Cuttings of 5-month-old Gorilla Glue (GG) mother plant were taken and rooted in an EZ-Cloner Classic ChamberTM (Sacramento, California) under aeroponic conditions. Rooted cuttings were grown under 18-h light/6-h darkness for 9 weeks in the closed-loop commercial facility, CTPharma LLC. Plants were then grown under 12 h light/12 h dark for reproductive growth.

Samples, Fig. 6: Cuttings of a 5-month-old Wife mother plant, grown at the University of Connecticut, were taken and rooted in Clonex rooting hormone (Hydrodynamics, MI, USA). Cuttings were grown under 18 h light/6 h dark. They were transplanted with Pro-Mix (AK, USA) Soilless mix. After 4 weeks in vegetative light, plants were grown under 12 h light/12 h dark for reproductive growth.

### Cloning of promoters and in silico promoter analysis

Promoters of *Cannabis* *sativa* *CsPT1* and *CsPT4* were identified using the reference genome information on NCBI. For *CsPT4* *pro*, we were able to clone 600 bp of the promoter, while we were able to clone 1500 bp of the *CsPT1* *pro*. The promoters were PCR amplified using iProof High Fidelity Polymerase (Bio-Rad, CA, USA). *CsPT1* *pro* and *CsPT4* *pro* were inserted into an entry vector pDONR221, and then cloned into a destination vector, either P2GWL7 (for luciferase assay) or pBGWFS7 (for GUS assay). The orientation of insertion is shown in Supplementary Fig. 1.

After the final insertion, plasmid maxiprep was conducted, with Macherey Nagel’s NucleoBond Xtra Maxi EF kit (Duren, Germany). *CsPT1* *pro* and *CsPT4* *pro* were analyzed using plant cis-acting regulatory DNA element databases, PLACE and PlantCARE. The predicted hormone responsive elements of *CsPT4* *pro* and *CsPT1* *pro* are shown in Supplementary Figs. 3 and 4 respectively.

### GUS assay

Cannabis seed sterilization and seedling growth was conducted according to Sorokin et al.^67^. After germination, seedlings were placed on full MS (pH 5.6) plates for 10 more days. Then the seedlings were used for transient expression. Leaves were taken from flowering Auto Tsunami plants, an auto-flowering variety, grown in a growth chamber with LED lights of 16 h light/8 h dark at 26 °C. Agrobacterium mediated transient gene expression in cannabis seedlings and leaves followed the method published by Deguchi et al.^68^. The Agrobacterium strain used was GV3101. GUS staining was performed according to the protocol in Arabidopsis: A Laboratory Manual^69^. GUS-stained seedlings and leaves were recorded using a Cannon digital camera.

### Hormone treatment of protoplasts and real-time quantitative PCR (qPCR)

Arabidopsis leaves were collected from wt plants of 3–4 weeks old. Two protocols were followed for protoplast isolation. The tape-sandwich method was followed directly to separate the lower epidermal layer of the leaves, exposing the mesophyll cells^70^. The remaining leaves were used to enzymatically isolate the protoplasts following a protocol published by Yoo et al.^71^.

Hormones were administered to Arabidopsis protoplasts at concentrations of 5, 10, and 50 µM, to determine the concentration that would be used in the dual luciferase reporter assay (DLR). These concentrations were shown to cause a response in Arabidopsis^72^. Seven hormones were administered to protoplasts, obtained from Sigma-Aldrich (MO, USA): cytokinin (trans-zeatin riboside, ZR), auxin (1-naphthaleneacetic acid, NAA), gibberellic acid 3 (GA)_,_ salicylic acid (SA), abscisic acid (ABA), ethylene (1-aminocyclopropane-1-carboxylic acid, an ethylene precursor, ACC), and methyl jasmonate (MeJA).

Representative hormone responsive genes were chosen for qPCR (Supplementary Table 1) to ensure the hormones were activating the signaling pathways required for DLR assays^72–75^. Protoplasts were treated with various concentrations of hormones, then collected after 4 h of shaking at 40 rpm in the dark, for RNA isolation and qPCR analysis.

Total RNA was isolated using TriReagent (Molecular Research Center, OH, USA) following the manufacturer’s protocol. cDNA was synthesized using iScript™ Reverse Transcription Supermix (Bio-Rad). qPCR was performed using iTaq Universal SYBR Green Supermix (Bio-Rad) in a CFX Connect system, with *AtGAPDH* as the housekeeping gene. qPCR program used: 95 °C for 3 min, followed by 39 cycles of 95 °C for 10 s, and 55 °C for 30 s. Primers are listed in Supplementary Table 2. The hormone concentration which caused the highest fold increase in the gene, relative to the control, was chosen for further promoter analysis and DLR (Supplementary Table 3).

### Dual luciferase reporter assay

Dual Luciferase Assays were conducted in which *CsPT1* *pro* and *CsPT4* *pro* were cloned upstream of the firefly LUC gene; CaMV 35S promoter driven *Renilla* LUC was used as control. Arabidopsis protoplasts were transfected with both constructs (10 μg firefly LUC plasmid, 10 ng *Renilla* LUC plasmid^76^). Relative luminescence is calculated by dividing the recorded firefly luminescence by the *Renilla* luminescence. An empty P2GWL7 plasmid was also transfected as a negative control, which ensured there was no background noise.

Following overnight transfection, the protoplasts were treated with hormones. *CsPT4* *pro* received 10 µM ZR, 10 µM ACC, 50 µM SA, 50 µM GA, 10 µM NAA and 10 µM ABA. *CsPT1* *pro* received 50 µM GA, 50 µM SA, 10 µM NAA, 10 µM MeJA and 10 µM ABA. Samples were collected at 1, 3, and 5 h post treatment (hpt). ZR, SA, GA, and ABA were dissolved in DMSO, while ACC, NAA, and MeJA were dissolved in DI water. The control samples were administered equal proportions of the solvent.

DLR reporter assay system was used (Promega, WI, USA) with the only alteration being the lysis buffer. Cell culture lysis reagent (Promega) was used instead of passive lysis buffer. A CLARIOstar Plus plate reader (BMG Labtech, Germany) was used. Statistical analysis was determined using a one tailed Student *t*-test. The experiment was repeated three times.

### Site-directed mutagenesis

Site directed mutagenesis was conducted on the SA responsive element (SARE) in the cloned region of the *CsPT4* *pro*. Q5 site directed mutagenesis kit (NEB, MA, USA) was used in which point mutations were made to the sequence of the putative SARE. See Supplementary Table 2 for primer design and Supplementary Fig. 5 for sequence comparison. Transfection was repeated with the mutated promoter, and luminescence was measured following SA treatment as mentioned above.

### RNA isolation from cannabis tissues and qPCR

Approximately 250 mg of tissue was collected from the cannabis plants. Samples from GG included root, leaf, petiole, and flower. Samples from Wife were flowers. Tissues were flash frozen with liquid nitrogen and stored at − 80 °C. Total RNA was isolated from samples using Plant and Fungi RNA Extraction Kit (Macherey Nagel, Duren, Germany). Single-stranded cDNA was synthesized as described above.

Primers for qPCR are listed in Supplementary Table 2. *CsPP2A* was used as the housekeeping gene for Wife samples, with 4 biological replicates for each treatment. *CsUbiquitin* was used for GG samples, with 3 biological replicates for each organ from the plant. qPCR was conducted as stated previously, with same polymerase and program. Samples were analyzed using the delta delta ct method and an ANOVA was conducted using a paired Student’s *t*-test.

Root Drench: SA was dissolved in 1 mL of DMSO and further diluted in 1 L of DI water, to obtain a 1 mM concentration. There were 4 biological replicates per treatment group. DMSO (control) or SA was administered to the plants as a soil drench during the fourth week of flowering; treatment methods from other studies were followed^60,61^. Flower samples were collected at hour 0, 3, 6, 12, 24, 48, and 72 h post treatment. Approximately 250 mg fresh flower samples were collected and flash frozen for qPCR assay.

### MeSA treatment and HPLC analysis

Floral Spray: MeSA (Sigma-Aldrich) was dissolved in 1 mL of DMSO, then diluted in 1 L DI water to create a concentration of 100 µM. DMSO or MeSA was applied by foliar spray until flowers were fully soaked; treatment methods from other studies followed^77,78^. The spray was applied during the fourth week of flowering, as a one-time treatment. Flower samples were collected from plants prior to treatment and two weeks post treatment. Cannabinoids were sampled by milling the entirety of each plant’s flowers into a composite mixture. Approximately 500 mg of flower tissue was used for cannabinoid extraction using 20 mL 9:1 methanol:dichloromethane (DCM). Samples were shaken for 1.5 h, then discarded. Samples were then diluted with 100 µL extraction to 900 µL 9:1 methanol:DCM.

Cannabinoid concentration was analyzed on a Shimadzu High Pressure Liquid Chromatograph (HPLC) Instrument (LC-10). The column used was a Raptor Arc C18 (150 × 4.6 mm, 2.7 μm particle size) (Restek, PA, USA). The HPLC method followed Shimadzu’s protocol^79^. Peaks on the chromatogram were identified using cannabinoids standards (Restek, PA).

### Statement of experiential plant materials

All procedures were conducted in accordance with the guidelines. Permissions or licenses were obtained to collect the plant materials.

## Supplementary Information

Supplementary Information.

## Data Availability

All relevant data can be found within the manuscript and its supporting materials.
