# Supplementary material for: Hormonal control of promoter activities of Cannabis sativa prenyltransferase 1 and 4 and salicylic acid mediated regulation of cannabinoid biosynthesis
Source: Sci Rep. 2023 May 27;13:8620. doi: 10.1038/s41598-023-35303-4 (PMC10224980; doi:10.1038/s41598-023-35303-4)
Supplement: Supplementary file 1 — Supplementary Information. [file 41598_2023_35303_MOESM1_ESM.pptx]

## Slide 1
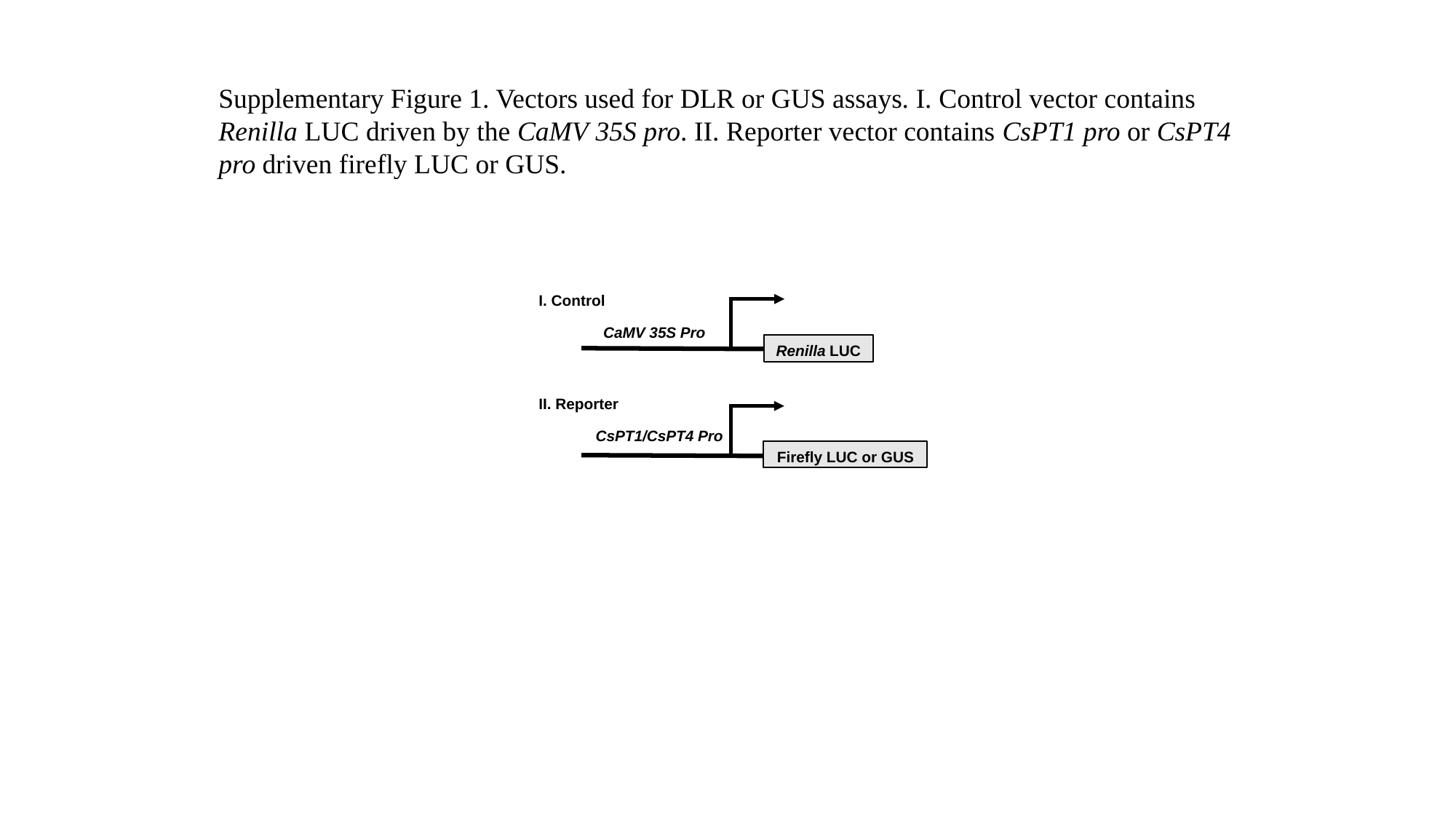

Supplementary Figure 1. Vectors used for DLR or GUS assays. I. Control vector contains Renilla LUC driven by the CaMV 35S pro. II. Reporter vector contains CsPT1 pro or CsPT4 pro driven firefly LUC or GUS.
I. Control
CaMV 35S Pro
Renilla LUC
II. Reporter
CsPT1/CsPT4 Pro
Firefly LUC or GUS

## Slide 2
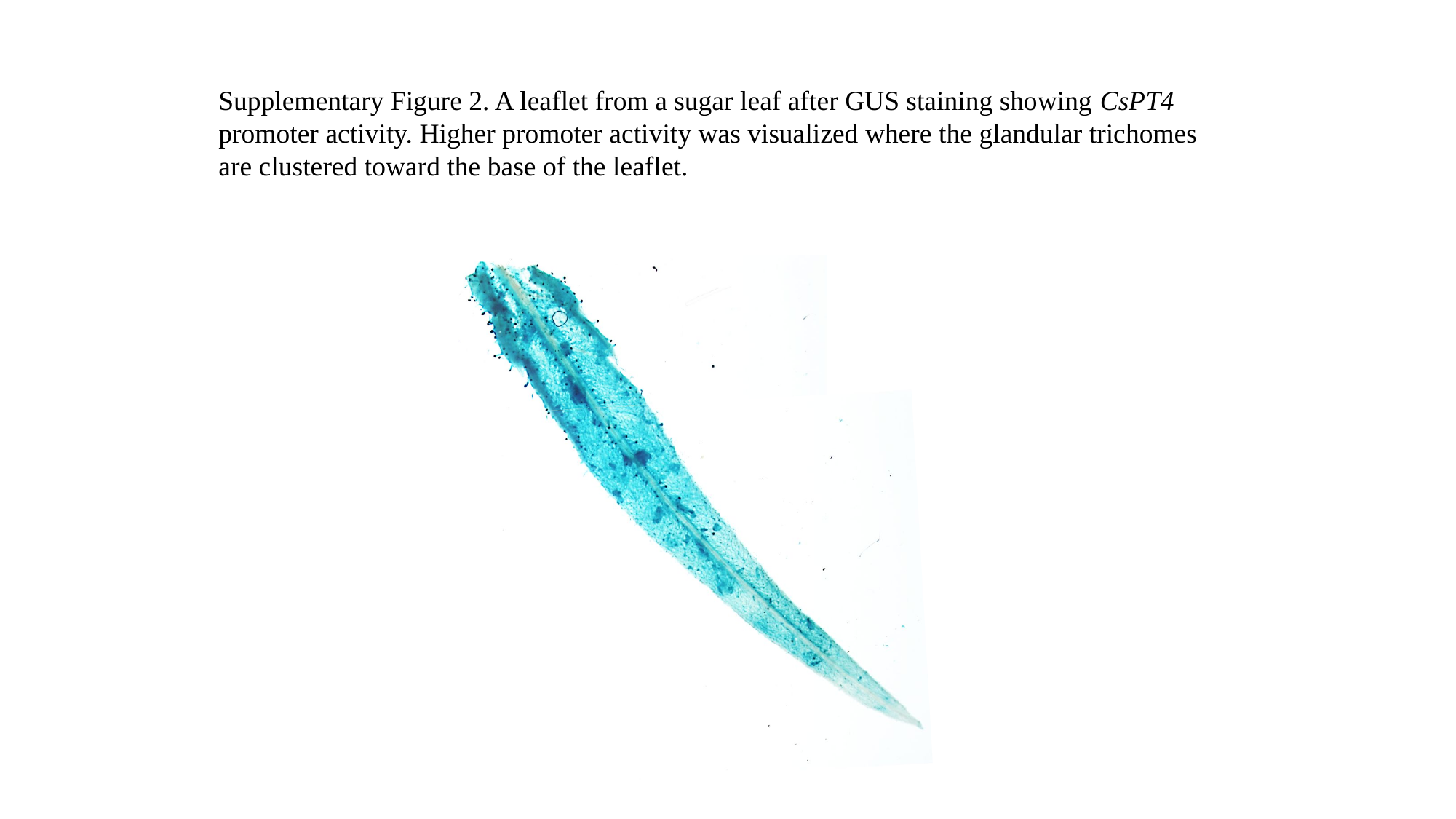

Supplementary Figure 2. A leaflet from a sugar leaf after GUS staining showing CsPT4 promoter activity. Higher promoter activity was visualized where the glandular trichomes are clustered toward the base of the leaflet.

## Slide 3
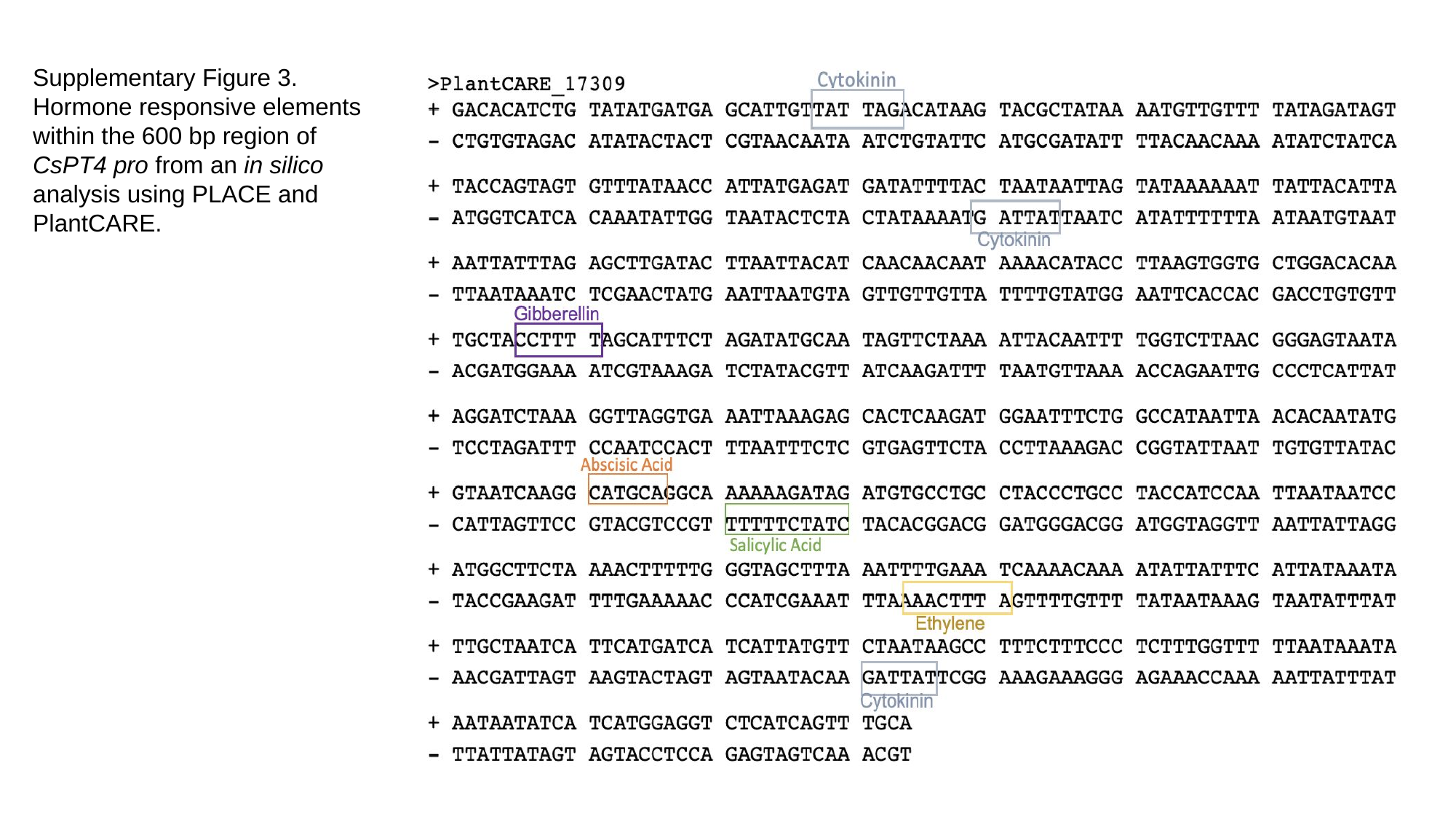

Supplementary Figure 3. Hormone responsive elements within the 600 bp region of CsPT4 pro from an in silico analysis using PLACE and PlantCARE.

## Slide 4
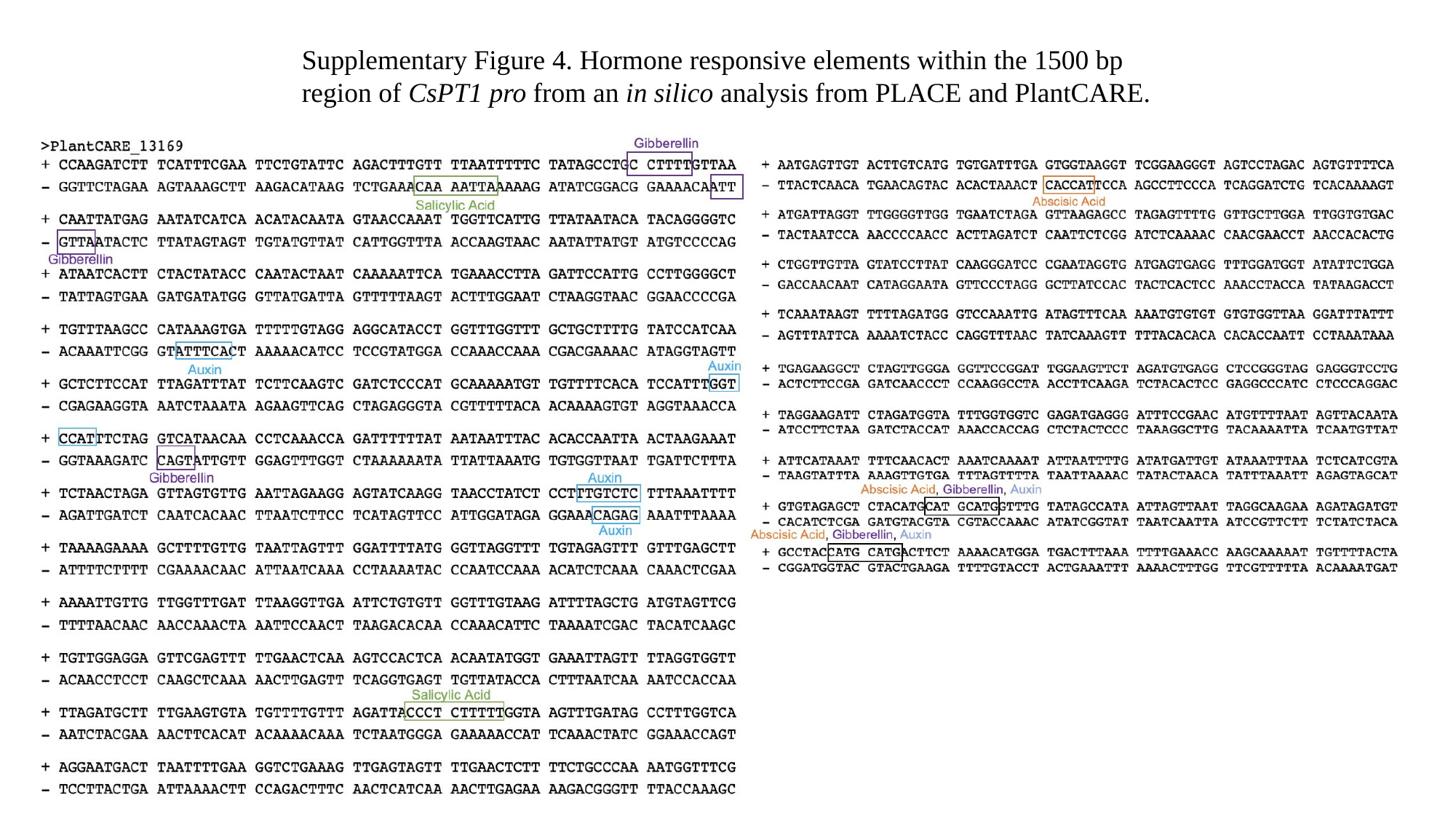

Supplementary Figure 4. Hormone responsive elements within the 1500 bp region of CsPT1 pro from an in silico analysis from PLACE and PlantCARE.

## Slide 5
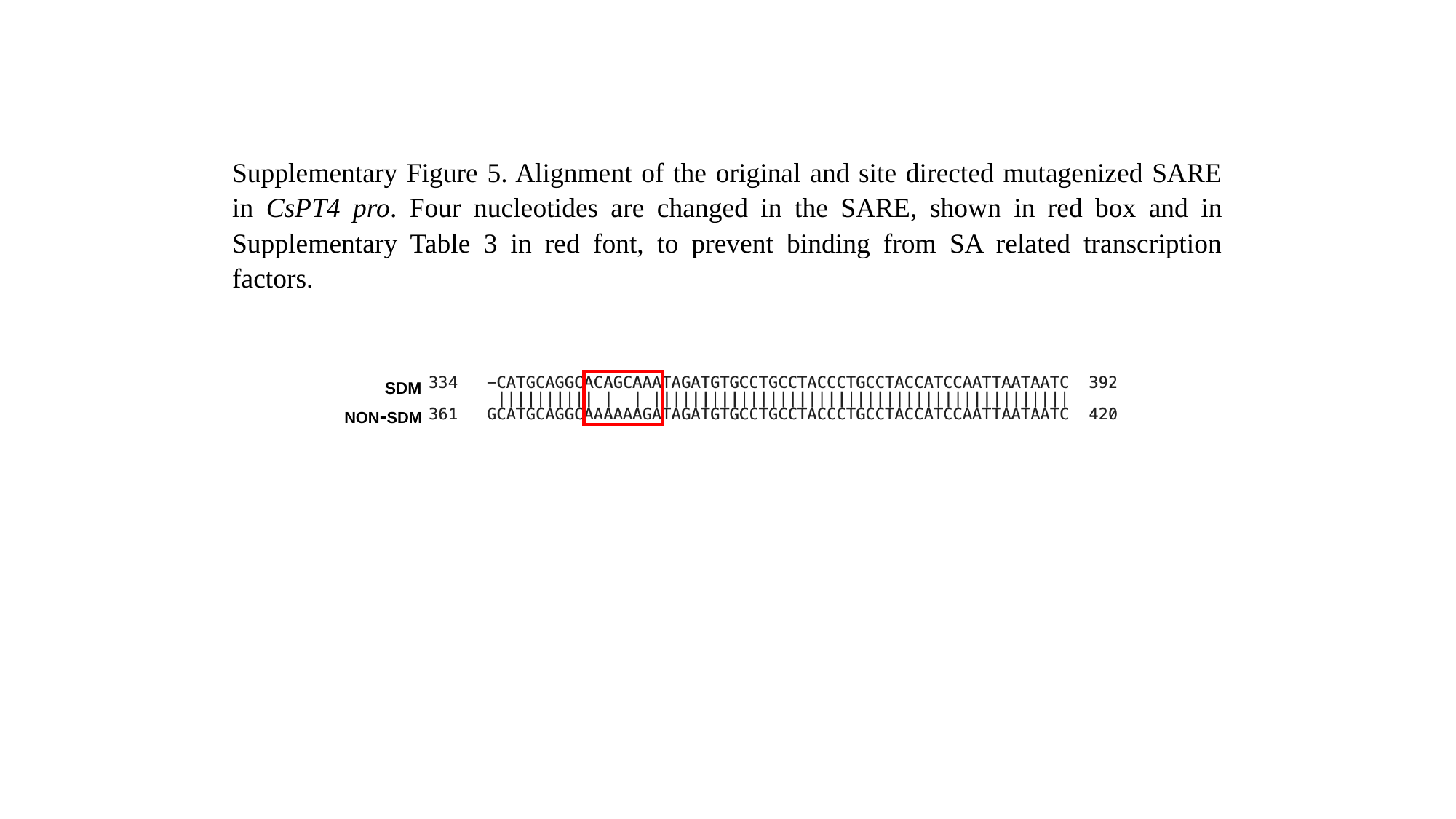

Supplementary Figure 5. Alignment of the original and site directed mutagenized SARE in CsPT4 pro. Four nucleotides are changed in the SARE, shown in red box and in Supplementary Table 3 in red font, to prevent binding from SA related transcription factors.
 SDM
NON-SDM

## Slide 6
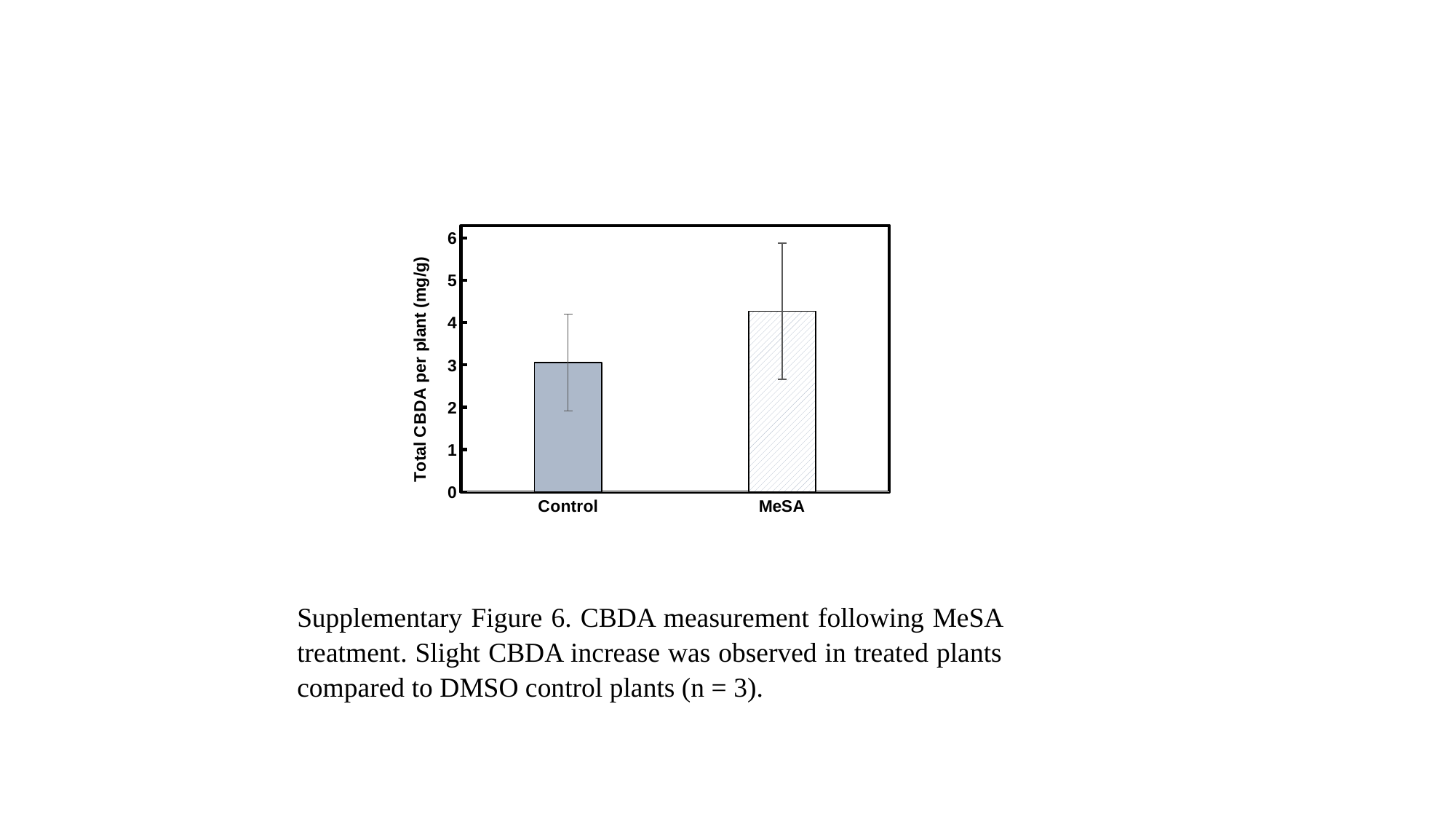

### Chart
| Category | Total CBDa Average (g/plant) |
|---|---|
| Control | 3.056288966666667 |
| MeSA | 4.274313500000001 |Supplementary Figure 6. CBDA measurement following MeSA treatment. Slight CBDA increase was observed in treated plants compared to DMSO control plants (n = 3).

## Slide 7
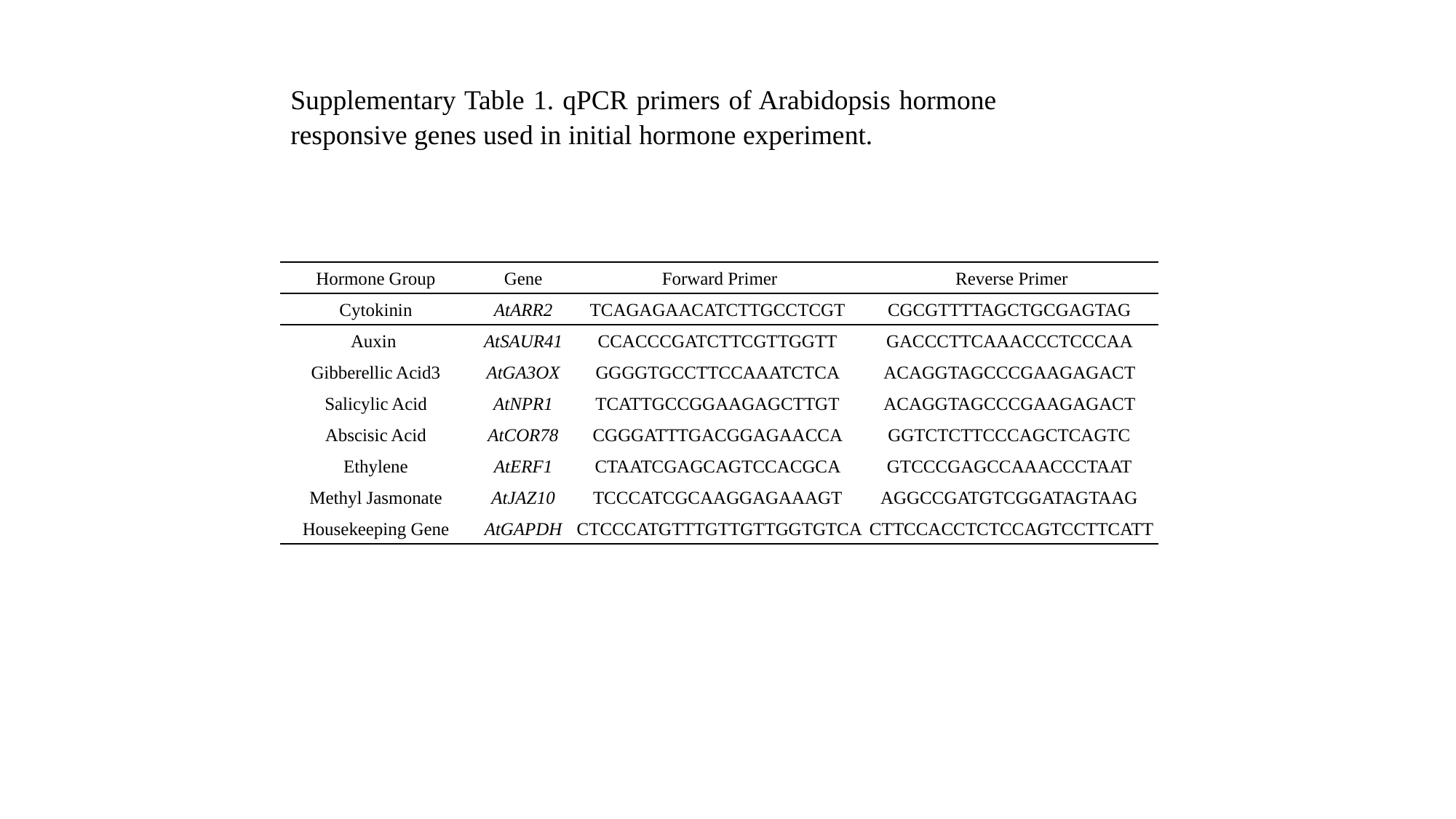

Supplementary Table 1. qPCR primers of Arabidopsis hormone responsive genes used in initial hormone experiment.
| Hormone Group | Gene | Forward Primer | Reverse Primer |
| --- | --- | --- | --- |
| Cytokinin | AtARR2 | TCAGAGAACATCTTGCCTCGT | CGCGTTTTAGCTGCGAGTAG |
| Auxin | AtSAUR41 | CCACCCGATCTTCGTTGGTT | GACCCTTCAAACCCTCCCAA |
| Gibberellic Acid3 | AtGA3OX | GGGGTGCCTTCCAAATCTCA | ACAGGTAGCCCGAAGAGACT |
| Salicylic Acid | AtNPR1 | TCATTGCCGGAAGAGCTTGT | ACAGGTAGCCCGAAGAGACT |
| Abscisic Acid | AtCOR78 | CGGGATTTGACGGAGAACCA | GGTCTCTTCCCAGCTCAGTC |
| Ethylene | AtERF1 | CTAATCGAGCAGTCCACGCA | GTCCCGAGCCAAACCCTAAT |
| Methyl Jasmonate | AtJAZ10 | TCCCATCGCAAGGAGAAAGT | AGGCCGATGTCGGATAGTAAG |
| Housekeeping Gene | AtGAPDH | CTCCCATGTTTGTTGTTGGTGTCA | CTTCCACCTCTCCAGTCCTTCATT |

## Slide 8
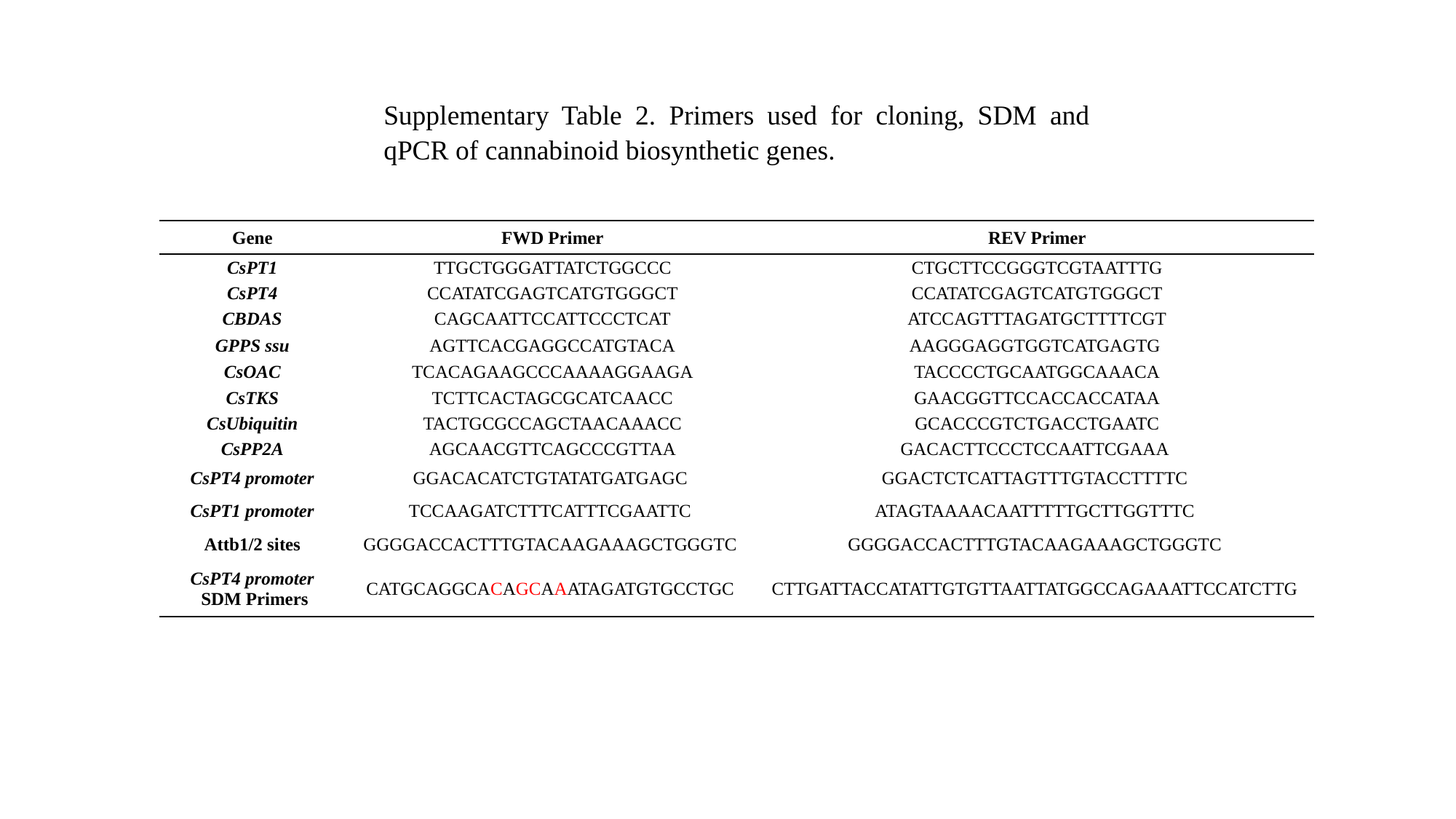

Supplementary Table 2. Primers used for cloning, SDM and qPCR of cannabinoid biosynthetic genes.
| Gene | FWD Primer | REV Primer |
| --- | --- | --- |
| CsPT1 | TTGCTGGGATTATCTGGCCC | CTGCTTCCGGGTCGTAATTTG |
| CsPT4 | CCATATCGAGTCATGTGGGCT | CCATATCGAGTCATGTGGGCT |
| CBDAS | CAGCAATTCCATTCCCTCAT | ATCCAGTTTAGATGCTTTTCGT |
| GPPS ssu | AGTTCACGAGGCCATGTACA | AAGGGAGGTGGTCATGAGTG |
| CsOAC | TCACAGAAGCCCAAAAGGAAGA | TACCCCTGCAATGGCAAACA |
| CsTKS | TCTTCACTAGCGCATCAACC | GAACGGTTCCACCACCATAA |
| CsUbiquitin | TACTGCGCCAGCTAACAAACC | GCACCCGTCTGACCTGAATC |
| CsPP2A | AGCAACGTTCAGCCCGTTAA | GACACTTCCCTCCAATTCGAAA |
| CsPT4 promoter | GGACACATCTGTATATGATGAGC | GGACTCTCATTAGTTTGTACCTTTTC |
| CsPT1 promoter | TCCAAGATCTTTCATTTCGAATTC | ATAGTAAAACAATTTTTGCTTGGTTTC |
| Attb1/2 sites | GGGGACCACTTTGTACAAGAAAGCTGGGTC | GGGGACCACTTTGTACAAGAAAGCTGGGTC |
| CsPT4 promoter SDM Primers | CATGCAGGCACAGCAAATAGATGTGCCTGC | CTTGATTACCATATTGTGTTAATTATGGCCAGAAATTCCATCTTG |

## Slide 9
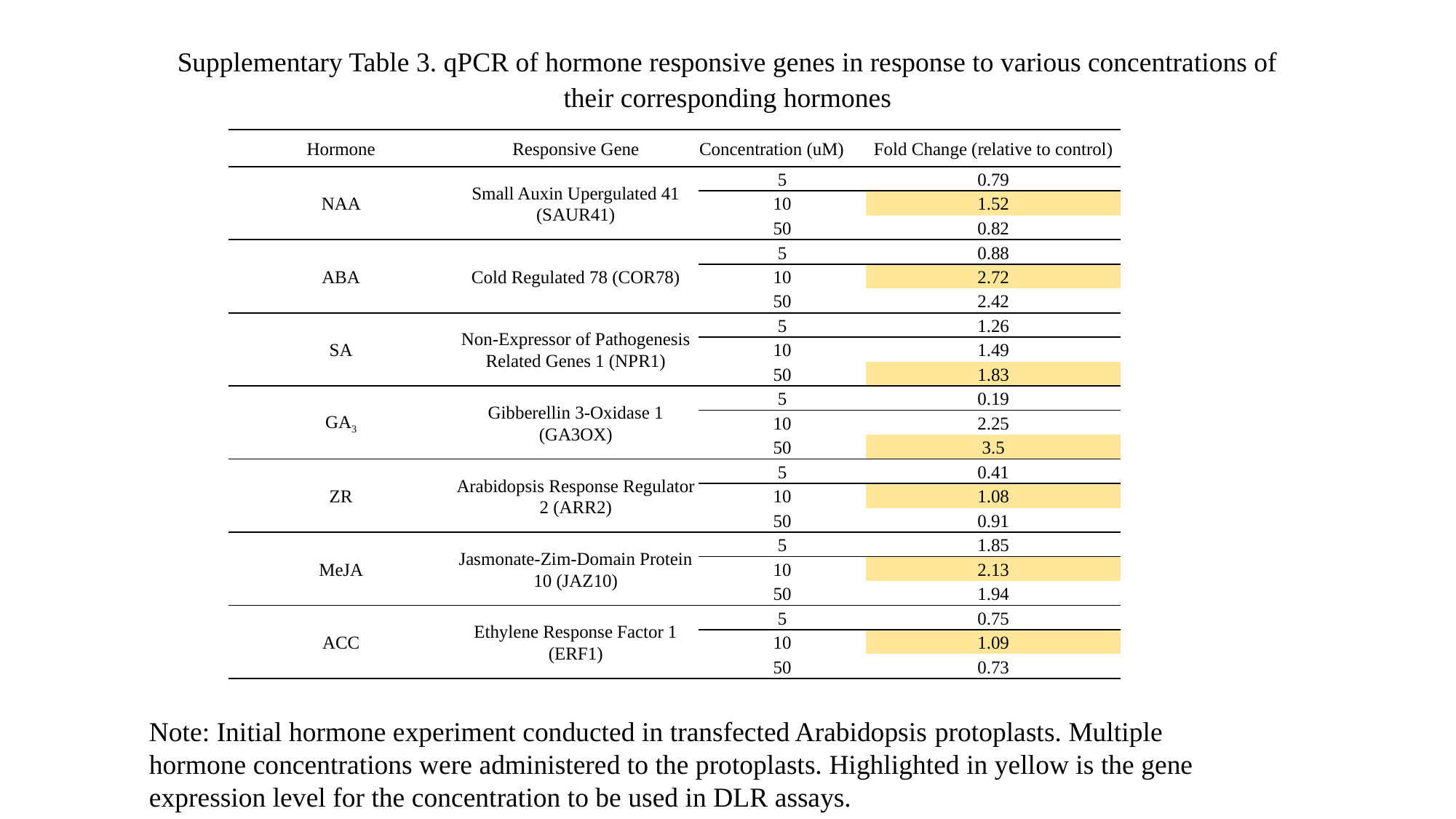

Supplementary Table 3. qPCR of hormone responsive genes in response to various concentrations of their corresponding hormones
| Hormone | Responsive Gene | Concentration (uM) | Fold Change (relative to control) |
| --- | --- | --- | --- |
| NAA | Small Auxin Upergulated 41 (SAUR41) | 5 | 0.79 |
| | | 10 | 1.52 |
| | | 50 | 0.82 |
| ABA | Cold Regulated 78 (COR78) | 5 | 0.88 |
| | | 10 | 2.72 |
| | | 50 | 2.42 |
| SA | Non-Expressor of Pathogenesis Related Genes 1 (NPR1) | 5 | 1.26 |
| | | 10 | 1.49 |
| | | 50 | 1.83 |
| GA3 | Gibberellin 3-Oxidase 1 (GA3OX) | 5 | 0.19 |
| | | 10 | 2.25 |
| | | 50 | 3.5 |
| ZR | Arabidopsis Response Regulator 2 (ARR2) | 5 | 0.41 |
| | | 10 | 1.08 |
| | | 50 | 0.91 |
| MeJA | Jasmonate-Zim-Domain Protein 10 (JAZ10) | 5 | 1.85 |
| | | 10 | 2.13 |
| | | 50 | 1.94 |
| ACC | Ethylene Response Factor 1 (ERF1) | 5 | 0.75 |
| | | 10 | 1.09 |
| | | 50 | 0.73 |
Note: Initial hormone experiment conducted in transfected Arabidopsis protoplasts. Multiple hormone concentrations were administered to the protoplasts. Highlighted in yellow is the gene expression level for the concentration to be used in DLR assays.
